# Freeze-drying protocols and methods of maintaining the in-vitro biological activity of horse platelet lysate

**DOI:** 10.1080/23144599.2024.2380586

**Published:** 2024-08-07

**Authors:** Chiara Bernardini, Noemi Romagnoli, Isabelle Casalini, Maria Elena Turba, Alessandro Spadari, Monica Forni, Fabio Gentilini

**Affiliations:** a Department of Veterinary Medical Sciences, University of Bologna, Ozzano dell ’Emilia, Bologna, Italy; b Health Sciences and Technologies-Interdepartmental Center for Industrial Research (CIRI-SDV), Alma Mater Studiorum—University of Bologna, Bologna, Italy; c Genefast srl, Forlì, Italy; d Department of Medical and Surgical Sciences, University of Bologna, Bologna, Italy

**Keywords:** Mesenchymal stem cells, large animal, equine, platelet lysate

## Abstract

Platelet lysate, derived from platelets, are valuable biological products rich in bioactive molecules. Their use promotes tissue healing and modulates inflammation. However, maintaining the stability and bioactivity of platelet lysate is challenging since they degrade rapidly at room temperature. This study focused on the possibility to confer enhanced stability to freeze-dried equine platelet lysate as an alternative to platelet-rich plasma (PRP). Platelet lysate (PL) was derived from PRP and freeze-dried either as such or using various adjuvants. Primary cell cultures of porcine Vascular Wall-Mesenchymal Stem Cells were treated with different PL formulations, and cell viability was assessed using an MTT assay. Overall, the addition of PL significantly improved cell viability as compared to controls without growth factor supplementation or with foetal bovine serum. Notably, the freeze-drying process maintained the effectiveness of the PL for at least a week. Furthermore, the study revealed that varying the horse as the source of PL could yield varying effects on cell viability. Detailed freeze-drying protocols were established, including freezing, primary drying and secondary drying phases, and the type of adjuvant. This study demonstrated the potential of freeze-dried equine PL as a viable alternative to PRP and highlighted the importance of precise freeze-drying protocols and adjuvants for standardization. Equine PL showed promise for medical treatment in horses, offering advantages such as extended shelf life, ease of handling, and reduced transportation costs, with the potential for broadened therapeutic usage.

## 1. Introduction

The platelet lysate (PL) are biological products obtained from whole platelets or their content. They are produced by processing platelet concentrates sourced from peripheral blood using differential centrifugation or apheresis so that the concentration of the bioactive molecules is fold higher with respect to the whole blood [1,2]. Depending upon the production process, there are different formulations of PL, all characterized by growth factors, such as platelet-derived growth factor (PDGF), vascular endothelial growth factor (VEGF), transforming growth factor beta (TGF-β), epithelial growth factor (EGF), basic fibroblast growth factor (bFGF), connective tissue growth factor (CTGF); bioactive peptides, such as BMPs (bone morphogenic proteins), and cytokines. Overall, these bioactive molecules stimulate cell proliferation, matrix deposition, and angiogenesis, and therefore, promote tissue healing and modulate the inflammatory process [3].

Bioactive molecule concentration and activity in PL have to be preserved in order to guarantee their effectiveness since bioactive molecules degrade at room temperature, and they cannot be detected as early as 2 weeks [4]. Fresh PL may be appropriately frozen at very low temperatures, i.e. −80°C in order to maintain a stable preparation, enabling storage of multiple doses with a single blood collection [5]. To circumvent the needs of long-term cryopreservation, the difficulty in transporting frozen samples and the relative cost, the lyophilization technique was evaluated [4, 6–11]. Lyophilization is a drying method which increases the shelf life of therapeutic molecules and facilitates their packaging, preservation, and transport [12]. Lyophilization has recently been applied to PRP by means of the preparation of powder or wafers from the blood of healthy human patients [7,8]. Freeze-dried PL have many advantages, such as adequately preserving PRP bioactivity, enabling standardization of the biomolecule concentration, and facilitating their handling and long-term preservation [2,4,6–8] Moreover, lyophilization preserves the antibacterial property of PL [8,13].

Of the various PL, platelet-rich plasma (PRP) is the most popular and utilizes intact platelets which are activated upon usage; PRP has been utilized in many medical scenarios, such as orthopaedics, odonto-stomatology, ophthalmology, and wound healing in humans [14]

The application of PRP has also been gaining widespread interest in equine veterinary practice. The effectiveness of PRP in horses has especially been described for tendon and ligament repair [15–21]; however, it can be utilized in numerous other situations. In particular, the authors observed that PRP promoted the proliferation and migration of equine corneal cells [22], it was a safe and effective aid for the treatment of osteoarthritis [23,24], and it reduced healing time and promoted the healing process when applied on skin wounds [25]. Nonetheless, a great variability in effectiveness has been reported which is likely associated with a lack of standardization in production and formulation [2]; [9]; [14]; [26–28]. Several methods have been described for preparing PRP in equine medicine [20]; [29]; [30]. Of the methods described, some [20]; [31] allow the preparation of multiple aliquots of PRP for multiple applications from a single blood collection which needs to be kept frozen at − 80°C. However, the cold chain for preservation and transport can be difficult to be maintained for the equine private practitioner and this limits the application of PRP in equine medicine. However, activated PRP has a gel-like consistency which hampers its prompt dissolution and makes it difficult to be standardized [32–38].

As alternative to PRP, PL obtained from PRP, was used; PL is a blood product free of xenogenic substances since it is cell-free and devoid of cell membranes, and it is specifically suitable for *in vitro* studies [39,40].

It is still a matter of discussion which factors or combination of factors are exactly involved in biological PL properties; specifically, biomolecule concentration per se does not necessarily predict the in vivo effect of the PL [10,28,41]. It has been observed that the claims of the standardization of PL production and formulation presented in many studies should also include the standardization of the method used to assess their biological function [2].

Currently, the characterization of PL, either fresh, frozen or lyophilized, may be carried out using indirect or direct methods. Indirect methods are used to measure the number of platelets, the growth factors and also biomolecule concentration [7,9–11,42]. Alternatively, direct methods ignore the quantitative measurement of bioactive molecules and instead focus on the direct assessment of their biological effect, using *in vivo* or *in-vitro* systems [11,42,43]. In particular, the beneficial effects of PL on mesenchymal stem cell cultures, were demonstrated [39,44].

The aim of this proof-of-concept study was to assess the ability of equine freeze-dried PL to maintain mesenchymal cell viability using a standardized and reproducible lyophilization protocol as compared to its fresh or frozen counterpart.

## 2. Methods

### 2.1. Experimental layout

**Figure 1.**
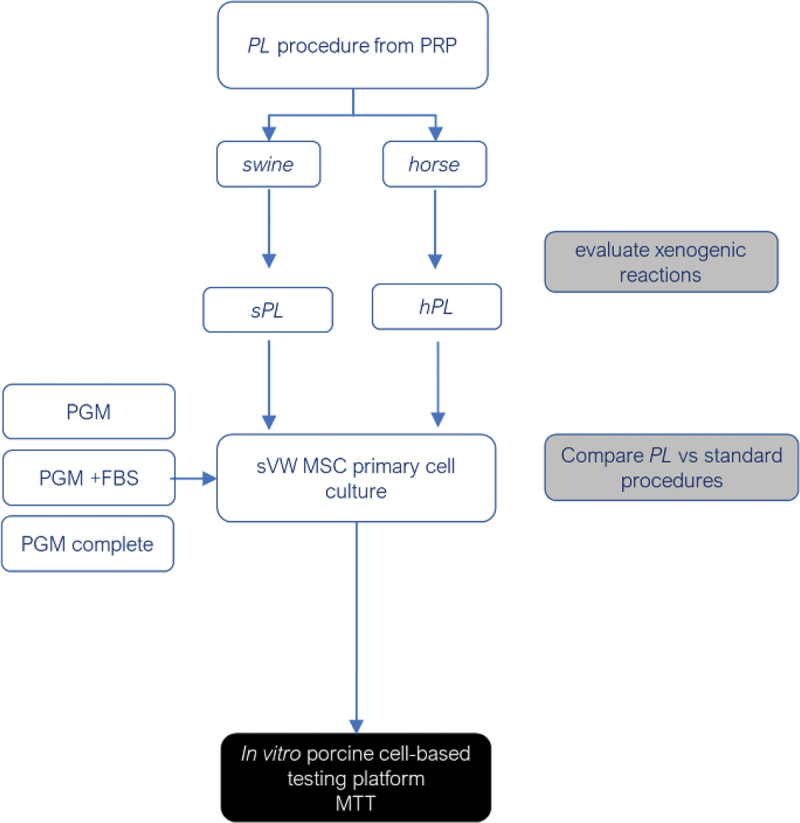
Schematic representation of the experimental workflow to exclude axenogenic effect: *hPL*: platelet lysate serum of the horse; *sPL*: platelet lysate serum of swine; HI: heat inactivated at 56°C for 30 min; sVW MSCs: swine vascular wall mesenchymal stem cells; RT: room temperature.

**Figure 2.**
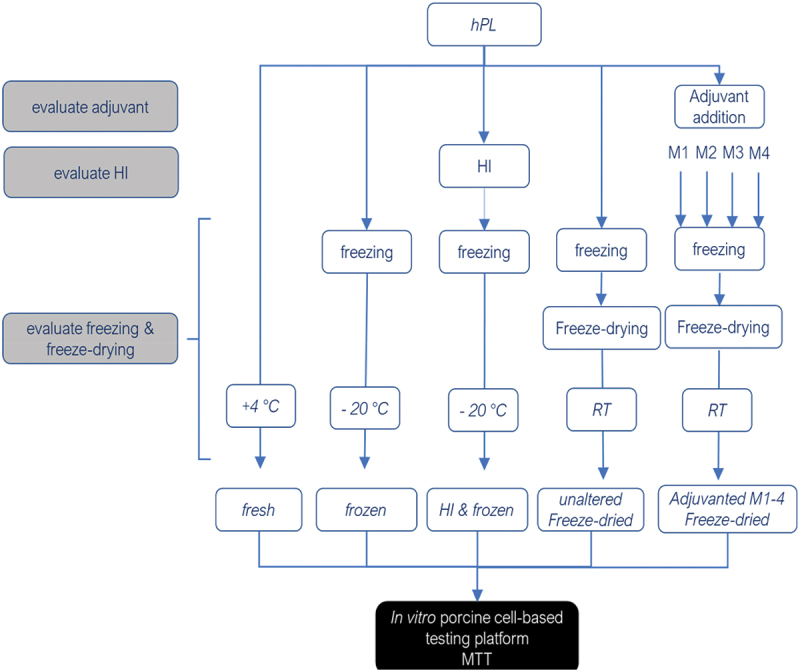
Schematic representation of the experimental workflow to test freeze-dried equine platelet lysate properties. *hPL*: platelet lysate of the horse; HI: heat inactivated at 56°C for 30 min RT: room temperature. M1: lyophilization mix 1, trehalose dihydrate 5% final concentration; M2: lyophilization mix 2, polyvinylpyrrolidone 40 5% final concentration; M3: lyophilization mix 3, D-Lactitol monohydrate 5% final concentration; M4: lyophilization mix 4, Glycin 2.5% and trehalose dihydrate 5% final concentration.

The possibility of the xenogenic adverse effects of equine PL on swine primary mesenchymal stem cell (MSC) cell culture was tested and excluded by comparing the viability of porcine Vascular Wall-Mesenchymal Stem Cells) (sVW-MSCs) cultured with Pericyte Growth Medium (PGM) to which swine or equine was added with sVW-MSC viability when cells were cultured with PGM alone (no growth factors added), or in PGM to which 10% Fetal Bovine Serum (FBS) was added or PGM to which vascular stem cell specific growth factors were added (Figure 1). The effects of the heat inactivation, the thermal treatment (fresh, frozen and freeze-dried) and the addition of adjuvants or not were then compared; the experimental layout is seen in a schematic graph in Figure 2.

### 2.2. Platelet rich plasma

Platelet-rich plasma was obtained utilizing the buffy coat method, using a 450-mL double transfusion bag (Terumo BCT, Rome, Italy). The blood was collected from the jugular vein from two healthy horses and two healthy swine, and was adequately mixed with citrate-phosphate dextrose solution with adenine (CPDA-1; 63 mL for 450 mL of blood). The separation of the blood cells was carried out using a refrigerated laboratory centrifuge (Heraeus Cryofuge 6000). Centrifugation was first carried out at 800 rpm for 20 min at an environmental temperature (2°C). The first centrifugation step led to the separation of the red and white blood cells from the plasma and platelets, separated by an intermediate thin greyish – white layer (buffy coat). The plasma component obtained was transferred to a satellite bag using a plasma extractor and subsequently underwent a second centrifugation at 3500 rpm for 10 min at an environmental temperature (22°C). After the second centrifugation, two components were obtained: PRP as a turbid fraction on the bottom of the bag, and another clear fraction above (platelet poor plasma PPP). The next step was the retransfer of the PPP into the initial bag containing the red blood cell component. The PRP was then separated under sterile conditions into different aliquots (1.8-mL Eppendorf vials; SARSTEDT, Verona, Italy) which were stored at −80°C.

### 2.3. PL

PL was produced from the PRP using the method described by Mojica-Henshaw *et al.* [39]. In more detail, frozen PRP was thawed at room temperature, then refrozen at − 80°C overnight, thawed again at + 4°C and then finally centrifuged at 4000 × g for 20 min. After the centrifugation, a sediment composed of cell membranes was evident at the bottom of the tubes while the supernatant was the PL-plasma. To precipitate the clotting factors, 100 µL of CaCl_2_ 2.5 M was added to approximately 1000 µL of PL-plasma (1:10 v/v) and left at 4°C overnight. The sample was then centrifuged at 4000 × g for 20 min; the fibrin clot was then mechanically detached using a sterile plastic loop and centrifuged again for 1 min at 11,000 × g. The supernatant PL was collected and transferred to a sterile tube until needed.

### 2.4. Adjuvants

Different adjuvants at 5% v/v final concentration, when not otherwise specified, were used to freeze-dry the PL samples, namely Trehalose (Trehalose dihydrate; lyophilization mix 1 (M1)), Polyvinylpyrrolidone 40 (PVP40; lyophilization mix 2 (M2)), Lactitol (D-Lactitol monohydrate; lyophilization mix 3 (M3)) and a mix of Glycin 2.5% and Trehalose (lyophilization mix 4 (M4)). All the reagents were purchased as powder from Sigma-Aldrich (Merck), solubilized in DHPLC grade water using a heated magnetic stirrer and sterilized by 0.22 µM filtering. The concentration of the stock solutions was 30% for Trehalose and Lactitol and 10% for Glycin and PVP40. The stock solutions were stored at −20°C until needed.

### 2.5. Freeze-drying

One mL of liquid PL with formulation, including or not adjuvants, was put into 5 mL glass vials and partially closed with lyophilization stoppers. Lyophilization was carried out using a freeze-dryer (VirTis Advantage Pro serie SP SCIENTIFIC) with two drying shelves and a pneumatic stoppering system to stopper the vials at the end of the lyophilization process. A preliminary freeze-drying study (not reported) carried out using a small amount (50 µL) of PL in lyophilization plastic tubes was used to establish an effective PL freeze-drying protocol and to reduce the adjuvant candidates to those most effective for that purpose.

The freeze-drying cycle, which included the three main freeze-drying stages: freezing, primary drying and secondary drying, was set up on the freeze-dryer and carried out in the following sequential order: initial stabilization phase at 8°C for 10 min, ramp shelf temperature (Ts) at − 40°C at max speed and held for 150 min at 560 Torr (Thermal treatment), the shelf temperature was brought to −25°C in 120 min and the pressure was lowered to 200 mTorr and held for 720 min, the shelf temperature was brought to −15°C in 120 min and the pressure was lowered to 200 mTorr and held for 360 min, the shelf temperature was brought to 0°C in 120 min and the pressure was lowered to 100 mTorr and held for 720 min, and then the shelf temperature was brought to 20°C in 360 min and the pressure was lowered to 50 mTorr and held for 720 min (drying steps). At the end of the programme, the lyophilization chamber was filled with NO_2_ progressively increasing the pressure to 760 Torr, and the vials were stoppered.

The freezing programme was shared by both samples, one to be conserved frozen and one to be freeze-dried. At the end of the freezing protocol, the samples treated by only freezing were stored at −20°C until use while the samples to be freeze-dried continued through the other two stages of the lyophilization programme.

### 2.6. Cell culture and treatments

Primary cell cultures of the VW-MSCs were previously isolated, recovered and characterized as described [45]. The VW-MSCs were thawed, seeded and routinely recovered in primary culture flasks (1.5 × 10^6^ cells/T75-flask) in PGM (Promocell, Heidelberg, Germany) to which 1× antibiotic-antimycotic solution and vascular stem cell specific growth factor mix (Promocell) was added to constitute the complete PGM medium. The cells were cultured in a 5% CO_2_ atmosphere at 38.5°C. The pVW-MSCs were thawed at the second pass age and grown in T-75 for three days; once they reached confluence, they were washed with Dulbecco’s Phosphate Buffered Saline (DPBS) (Gibco-Life Technologies, Carlsbad CA, USA), detached with 0.25 × Trypsin (Gibco-Life Technologies), counted using a Burker chamber and were seeded in 5 × 103/wells in a 96-well plate. The day after, the cells were treated under different conditions (Figure 1) for 24 hours. For the viability determination, an MTT-based assay (In Vitro Toxicology Assay Kit MTT TOX-1, Sigma-Aldrich St. Louis, MO, USA) was carried out at the end of the treatment time, following the manufacturer’s instructions. Briefly, the MTT substrate was added to the culture medium and incubated for 4 hours; the MTT solubilization solution was then added to dissolve the formazan crystals. Formazan Abs was measured at 570 nm, using Infinite® F50/Robotic absorbance microplate readers from TECAN Life Sciences (Männedorf, Switzerland). All the experiments were repeated three times independently; 8 technical replicates were used for each treatment.

### 2.7. Statistical analysis

The effect of the following factors was compared using the Kruskal-Wallis test for inequality of medians: the effect of different additions to the PGM (no addition, FBS, commercial addition or PL (either swine and equine), thermal treatment (fresh, frozen, or freeze-dried), horses used as the source of PRP, and the effect of the freeze-drying adjuvants. Estimations of the median differences between the groups were carried out using the Hodges-Lehmann location shift and were evaluated using the Steel-Dwass-Critchlow-Fligner pairwise ranking nonparametric method. Statistical significance was set at 5% (*p* < 0.05). All the analyses were carried out using the Analyze-it package software (Analyse-it Software, Ltd, UK).

## 3. Results

**Figure 3.**
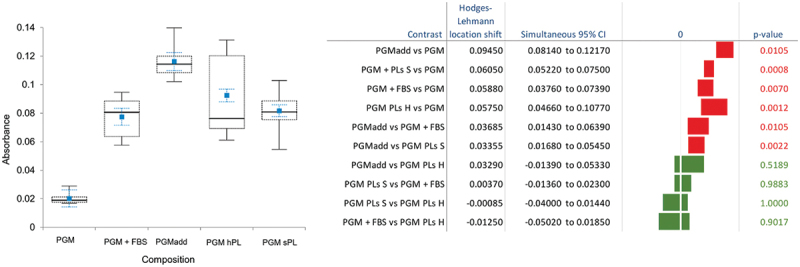
Skeletal Box plot representation of cell viability. The sVW-MSCs were cultured in PGM without growth factor supplementation (PGM), with 10% FBS (PGM +FBS), with MSC specific growth factors (PGMadd), with equine *PL* (PGM PL H), or with swine *PL* (PGM PLs S). The median is plotted in black as a line, the 1^st^ and 3^rd^ quartiles as a dashed box, and the maximum and minimum values as whiskers with end caps. The mean values ± SE (standard error) are reported in blue. FBS: fetal bovine serum; PGM: perycite growth medium; *PLs* H: platelet lysate serum of the horse: *PLs* S: platelet lysate serum of swine. Pair comparisons with a significance level using the Steel-Dwass-Critchlow-Fligner method and median difference estimations are indicated on the right.

No xenogenic reactions which could have hampered the effect of the PGM addition to the PL from the horses on the pVW-MSC culture were detected; in fact, no adverse effect on cell viability was observed with the addition of equine or swine PL (Figure 3).The addition of the specific commercial concentrates of the growth factors optimized for the growth of these vascular mesenchymal stem cells (PGMadd) achieved the best effect on cell viability, being significantly better than PGM alone, also against the addition of the common use of 10% FBS but not of equine *PL* which guaranteed high viability (Figure 3).

In the second experiment, it was confirmed that the two horses used as sources of PL, although they were not the same as those of the first experiment, yielded significant differences in the MTT assay (median 0.11780 vs 0.09780; p = 0.0001), red: *p* value < 0.05; green: *p* value > 0.05

**Figure 4.**
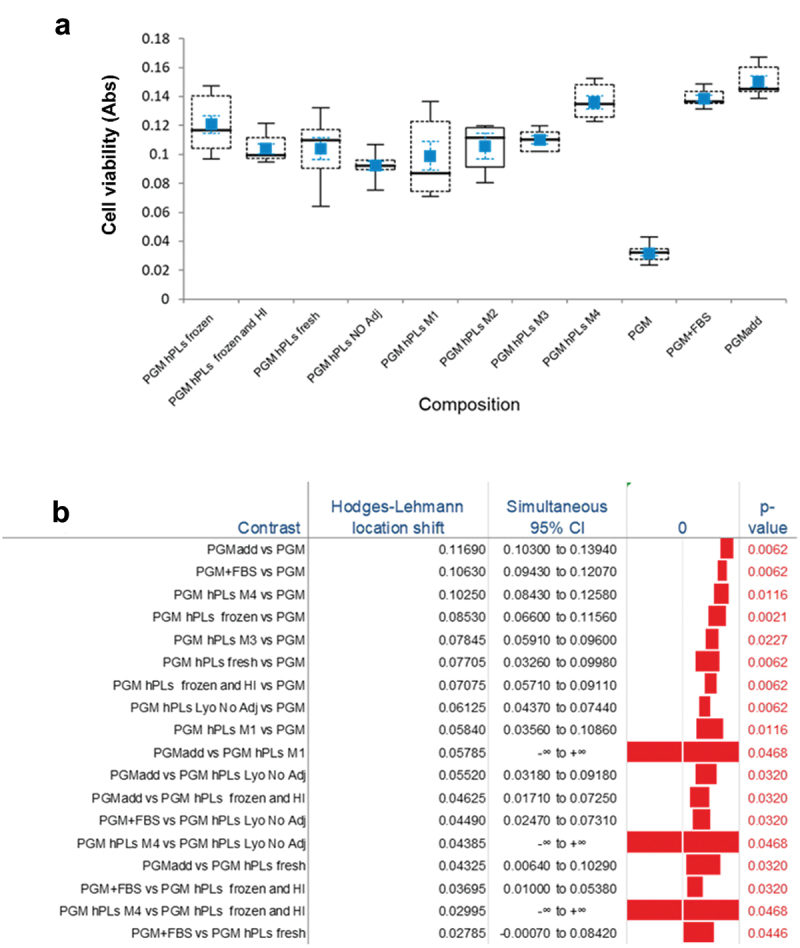
a) Skeletal Box plot representation of cell viability. The pVW-MSCs were cultured in PGM supplemented with equine *PL*, either frozen (PGM *hPLs* frozen), heat inactivated and then frozen (PGM *hPL* frozen and HI), freshly prepared and kept at + 4°C (PGM *hPLs* fresh), lyophilized without adjuvants (PGM *hPL* NO adj) or lyophilized using adjuvants (PGM *hPL* M1 to 4); PGM alone or PGM to which 10% FBS or commercial growth factors (PGMadd) were added were used as internal controls. The median is plotted as a line in black, the 1^st^ and 3^rd^ quartile as a dashed box, and the maximum and minimum values as whiskers with end caps. The mean values ± SE (standard error) are reported in blue. FBS: fetal bovine serum; PGM: perycite growth medium; *PLs* H: platelet lysate serum of the horse: *PL* S: platelet lysate serum of swine. Pair comparisons with a significance level using the Steel-Dwass-Critchlow-Fligner method and median difference estimations are indicated on the right. b) only statistically significant (*p* < 0.05) contrasts are reported.

In general, no toxic effect was evidenced: all the PL treatments and supplementations resulted in a significant increase in cell viability with respect to the PGM without growth factors (Figure 4). Moreover, the addition of PL was confirmed to significantly affect cell growth regardless of the treatments used to store the PL. The most effective addition was the use of a commercial brand growth factor supplement specific for MSCs which was also found to have a greater effect than the addition of either freeze-dried PL without adjuvants or that with adjuvants M1-M3, or heat inactivated and frozen, or fresh. On the contrary, adjuvant M4 reached results comparable with those of PGM add. Detailed pair comparisons findings are reported in Figure 4.

**Figure 5.**
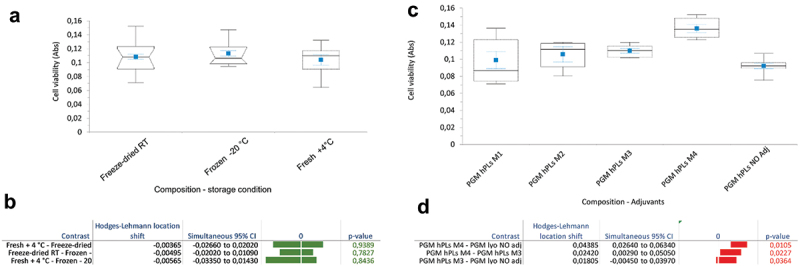
a) Skeletal Box plot representation of cell viability. The pVW-MSCs were cultured in PGM supplemented with the *PL*, either freeze-dried or frozen and kept at −20°C, or freshly processed and kept at +4°C, b) estimation of the median shift location and its significance. c) Skeletal Box plot representation of the comparisons of cell viability. The pVW-MSCs were cultured in PGM with different lyophilization mixes (PGM hPL M1-M4) or without mixes (PGM hPL NO adj) and c) estimation of the median shift location and its significance. Only statistically significant (*p* < 0.05) contrasts are reported. The median is plotted as a line in black, the 1^st^ and 3^rd^ quartiles as a dashed box, and the maximum and minimum values as whiskers with end ca. The mean values ± SE (standard error) are reported in blue. red: *p* value < 0.05; green: *p* value > 0.05.

The comparisons of the methods of storage of the PL, either freshly processed *PL* kept at + 4°C, frozen *PL* kept at −20°C or freeze-dried PL kept at room temperature for a week (Figure 5a, b) did not show any differences while it should be noted that M4 and M3 yielded a greater effect (Figure 5c, d).

## 4. Discussion

This study described a method of preparing a standardized freeze-dried equine PL, as an alternative to PRP, which had the same effectiveness in maintaining cell viability in vitro of the respective fresh and frozen equivalents, at least in the investigated short term of a week. This meant that freeze-drying would have no impact on the bioactive molecular content of the equine PL which, in turn, were, for the most past, expected to overlap with the spectra of the bioactive molecules involved in tissue reparation/regeneration *in vivo*. This proof-of concept study supported the possibility of extending this approach to *in vivo* studies and moved in the direction of a broader application of platelet lysate as therapeutics in horses. In fact, short-term conservation is appropriate for transporting the product to the site in which it is used without the need for maintaining a cold chain. This aim was reached regardless of whether or not adjuvants were used. The temperature-controlled shipment of biological materials is costly and demanding since it requires setting up dedicated supply chain of raw materials such as dry ice, and packaging, and complying with stringent laws and regulations regarding classifying, packing and labelling these shipments. The achievement herein described, by minimizing the need for a cold chain and lowering the relative costs and logistics, envisages in equine medicine a wider diffusion of the therapeutics applications of PL for treatments applied directly in the field almost everywhere.

A previous study reported significant changes in the concentrations contained in the frozen PL, limited to insulin-like growth factor 1 (IGF-1), when compared to fresh PDs [5]. Instead, another study reported a greater concentration of growth factors in lyophilized PRP as compared to fresh PRP since freeze-drying lyses the majority of platelets, releasing their granular content into the extracellular fluid for measurement. Regardless of the different concentrations of discrete biomolecules in fresh, frozen of freeze-dried PL, in almost all the studies, no differences were demonstrated in the biological effect in vitro or in vivo [2,7,10,11,42]. The effectiveness of freeze-drying in this context has notable supporting evidence which was not surprising [11]. However, it should be emphasized that some authors who have reported great variability in effectiveness have also referred to difficulties in standardizing lyophilization [14,46]. In fact, the freeze-drying protocols are crucial to keeping the desired qualitative features of PL. Unexpectedly, by reviewing the literature, it emerged that many studies were carried out using basic freeze-drying protocols and instruments without strict control over temperature and pressure, or without the possibility of automatically modifying parameters during the treatments as in advanced freeze-dryers. Likewise, detailed freeze-drying steps were reported sporadically [6] whereas, in the majority of studies, the lyophilization protocol was not reported [2,4,7,10,11]. Consequently, it was not possible to standardize such a complicated procedure as the freeze-drying of biologicals and, even more important, it was not possible to upgrade the process from experimental laboratory procedures to manufacturing for clinical applications [47,48]. For that reason, in this study, the Authors reported a detailed freeze-drying protocol which included all the typical phases of lyophilization: freezing (solidification), primary drying (ice sublimation) and secondary drying (moisture desorption) so that the entire cycle could be reproduced elsewhere. Tight control of the different phases of a freeze-drying cycle is crucial for guaranteeing its reproducible effectiveness [49,50]. In particular, when dealing with injectable drugs, such as biologicals, the gross appearance of the product (cake) is a relevant quality attribute, and a uniform and elegant cake is the main goal of an adequate lyophilization process. To that aim, the freezing phase affects the texture of the cake and, eventually, the aspect and the shelf-life of the products [49,50]. Controlling the freezing step accounts for ramp speed, and the lowest temperature achieved and its duration [50]. In this study, these parameters were used to freeze-dry a volume of product which resembled the one used in clinical application. This was because the volume of liquid determines the thickness of the cake which, in turn, affects the lyophilization process. Generally, the thicker it is, the more freeze-drying is hindered. Furthermore, adjuvants can be used to facilitate the cake formation. In the lyophilization of PL, the use of adjuvants has been evaluated in some studies [6,8,9] while, in other studies, the PL was freeze-dried as such [2,4,7,10]. The adjuvants included Sodium carboxymethylcellulose [8], Tris, glycine and sucrose solution [9] and HEPES, NaCl, KCl, MgCl_2_, trehalose, and human serum albumin [6]. In the present study, four lyophilization mixes were evaluated in a dozen different molecules after preliminary empirical screening. A mix of glycine and trehalose gave the best results but lactitol also showed a significantly positive effect. Trehalose may be crucial for maintaining the platelets intact during freeze-drying [32] which is not the case for PL in which the platelets are thoroughly lysed and their content completely released and, hence, in short-term conservation, the benefit of using adjuvants was not obvious. However, sugar and glycine also have a positive impact on better cake formation, and their use would be warranted in the case of the long-term conservation of PL [6,9,12].

In this study, straightforward evidence of the activity of equine PL on cell cultures obtained from distinct species was demonstrated. No evidence of xenogenic reactions was observed in-vitro which, however, needs to also be confirmed in vivo. Moreover, it is worth noting that no differences were observed when heat inactivation was used to inhibit the complement-mediated immune reactions. Heat inactivation may also lead to subtle damage of useful biomolecules apart from the complement [51]. The fact that heat inactivation of the PL is not necessary may be relevant both for the application of PL as a medium supplement in cell culture but also in the perspective of allogenic formulations. In this instance, no xenogenic molecules, such as bovine thrombin, were used; instead, the Authors intended to evaluate calcium as a platelet activator which was confirmed to be simple and effective [6]. Additional studies are necessary to explore this interesting possibility.

Finally, it should be considered that a great interindividual variability exists in the biological effect induced by various PL obtained under the same conditions [2,31,52–55]. The present study also confirmed this circumstance; in fact, the PL obtained from different horses gave significantly different *in vitro* effects. However, in the case of allogenic treatment, individual variability was controlled by mixing PL from different subjects; this was unlikely to represent a limitation whereas, in the case of autologous treatment, this would be the case [2]. If so, it should be noted that freeze-dried PL can be reconstituted using a different amount of water with respect to the original amount and, hence, the concentration of bioactive molecules can be concentrated or diluted as needed [7,41].

## 5. Conclusion

This study showed that equine PL maintained cell viability in vitro, reflecting a high content of bioactive molecules and, in addition to medical treatment for horses, it could conveniently be used as a medium supplement in cell cultures as an alternative to FBS. Finally, this study confirmed that freeze-drying was effective in keeping the therapeutic potential of these biologicals intact and provided details regarding lyophilization protocols which could be of aid for additional efforts towards standardizations.

## 6. Ethics approval and consent to participate

The care and handling of the animals were in accordance with the provisions of European Economic Community Council Directive 86/609, adopted by the Italian Government (D.L. 27 January 1992 n 116) PROT. n. 43-IX/9

## 7. Consent for publication

Not applicable

## 8. Availability of data and materials

The authors confirm that the main data supporting the findings of this study are available within the article. The datasets used and/or analysed during the current study are available from the corresponding author on reasonable request.

## Abbreviations

PRP: platelet-rich plasma
pVW-MSCs: porcine Vascular Wall-Mesenchymal Stem Cells
PL: Platelet lysate
H: Horse
PDGF: Platelet-derived Growth Factor
VEGF: Vascular Endothelial Growth Factor
TGF-β: Trasforming Growth Factor
EGF: Epithelial Growth Factor
bFGF: Basic Fibroblast Growth Factor
CTGF: Connective Tissue Growth Factor
BMPs: Bone morphogenetic proteins
FBS: Fetal Bovine Serum
PGM: Pericyte Growth Medium
S: Swine
DPBS: Dulbecco’s phosphate-buffered saline
CPDA-1: Citrate-phosphate dextrose solution with adenine
IGF_1: Insulin-like growth factor 1
